# Scientific Items

**Published:** 1885-01-20

**Authors:** 


### SCIENTIFIC ITEMS.

**Blue Eyes Going Out.**—Darwinian speculations lead to some curious conclusions. For instance, M. Alphonse de Candolle, in a paper on "Heredity in the Color of the Eyes in the Human Species" (of which Science gives an abstract), predicts that blue eyes are destined to disappear, at least in Europe. In investigating the subject of heredity, it occurred to him that the color of the iris offered the best outward and visible sign. It is conspicuous : it cannot be masked by artifice. After early childhood it does not vary with age, as does the color of the hair ; and the character is on the whole distinct : for, according to him, there are only two sorts—black, or rather brown, eyes, and blue ; gray eyes being reckoned as mere varieties of the blue. From the working-up of the statistics, in part from series of observations made for the purpose, it appears, that, when both parents have eyes of the same color, 88.4 per cent. of the children follow their parents in this feature ; and, of the 11.6 per cent. of children born with eyes of other than the parental color, a part must be attributed to atavism, that is, to intermittent heredity. But the curious fact comes out, that more females than males have black or brown eyes, in the proportion, say, of 49 to 45, or of 41 to 39. Next it appears that, with different-colored eyes in the two parents, 53.9 per cent. of the progeny followed the fathers in being dark-eyed, and 55.9 per cent. followed their mothers in being dark eyed. An increase of 5 per cent. of dark-eyed in each generation of "discolorous" unions must tell heavily in the course of time. It would seem, that, unless specially bred by "concolorous" marriages, blue-eyed belles will be scarce in the millenium.—*Popular Science News.*

**The Mean Temperature of the World.**—The Journal of the Society of Arts in London gives the following interesting facts on this subject, with incidental reference to recent experiments in the production of artificial cold :

The Royal Meteorological Society indicates the mean temperature of the months of January and July, in London, for one hundred years. The lowest mean for January occurred in 1795, and was 25.5° F. ; the highest mean for January, 47°, in 1796. The lowest mean temperature for July was 55.5°, in 1816, and the highest, 68°, in 1778 and 1859. The society also marks, in a similar way, the mean annual temperatures, and the lowest and highest temperatures, for several important and well-known places. The mean annual temperatures in this interesting series of records most worthy of note are, for Ferozepore, in Punjaub, 95° ; Calcutta, 81° ; Hongkong, 75° ; Cairo, 71° ; Rome, 60° ; Constantinople, 58° ; Pekin, 53° ; St. Petersburg, 40° ; Hammerfest, 35° ; Fort Enterprise, 14° ; Boothia Felix—which upon this ground should surely be rather marked as "Infelix"—5° ; and Melville Island, zero. The highest temperature recorded for the several stations are, for Murauk, in Fezzan, 135° ; Bagdad, 120° ; Cairo, 116° ; Jerusalem,

102°; Greenwich, 97°; Moscow, 90°; Falkland Isles, 75°. The lowest recorded are, for Barbadoes, 72°; Singapore, 60°; Bombay, 53°; Jerusalem, 25°; Constantinople, 17°; Greenwich, -10°; Chicago, -30°; Moscow, 53°; Melville Island, -65°; Fort Reliance, -70°; and Werschjansk, -81°, which outlandishly named place, so far, seems to possess the unenviable distinction of being the coldest spot upon the earth, unless a station nearer home may be inclined to contest with it the frozen supremacy upon the ground of an observation that has been made since these comparative records were drawn up. On the evening of the 13th of June last, in the lecture theater of the Royal Institution of Great Britain, in Albemarle street, a temperature of -252° F., with alcohol turned into a kind of viscid ice, and oxygen gas congealed into a liquid, was recorded. In this instance, however, it must, in meteoroligical fairness, be said, the result was brought about by human as well as by natural agency; the spell of boiling solid carbonic acid having been used in the final depression of the temperature.—*Ib.*

**Butter Fifty Years Old.**—A crock of butter was lately taken out of a well in New York State where it had lain fifty years. A Mrs. Jupp, who was a famous dairy woman, near Albany, half a century ago, used to lower her butter into a well on the premises which was noted for its very cold water. Mrs. Jupp would leave the butter hanging in the water for several hours, and when taken out it would be as hard and cold as ice. One day, in 1834, she was lowering a crock of butter into the well, when the rope broke, and the crock fell to the bottom. No effort was ever made to recover it. For the first time in its history this well became almost dry during the recent long drought. A few days ago the present owner was cleaning the well out, when he found the crock Mrs. Jupp had lost fifty years ago. In taking the crock from the well, the finder accidentally broke it. It was about one-quarter full of butter, which was as solid and sweet as it was the day it was put down, half a century ago.—*Pop. Science News.*

It is by no means probable that Neptune is the "outside" member of our planetary system; but, as in the case of Neptune, the existence and position of any more distant planet will very likely be figured out by the mathematician before the body is detected with the telescope. At a recent meeting of the French Academy of Sciences, M. Duponchel presented a paper on solar energy and the oscillations of the magnetic needle. He infers, from the observations made during the last three hundred years or more, that the secular variations of the needle are due to the action of an ultra-Neptunian planet having a period of about four hundred and sixty-seven years. He thinks the hypothetical orb is now in the longitude of 314° in the constellation Capricorn. Nature (to which we are indebted for this information) says that M. Duponchel proposes to call this planet "The Ocean;" but we suspect that the original paper says *Oceanus*, one of the most ancient of the Greek divinities, from whose name "Ocean" is derived. One can easily see how a careless translator might confound the two words in French, but that our learned contemporary should overlook the slip is passing strange.—*Ibid.*

